# 2392. Impact of Vaccination Against SARS-CoV-2 on Severity and Mortality in Hospitalized Patients with COVID-19 in a Referral Hospital in Nicaragua

**DOI:** 10.1093/ofid/ofad500.2012

**Published:** 2023-11-27

**Authors:** N O R V I N G VEGA-JARQUIN, Guillermo D Porras-Cortés

**Affiliations:** Hospital Dr. Fernando Vélez Paiz, Ciudad Darío, Matagalpa, Nicaragua; Hospital Dr. Fernando Vélez Paiz, Ciudad Darío, Matagalpa, Nicaragua

## Abstract

**Background:**

On October 2021, the World Health Organization exhorted countries to evaluate the effectiveness of vaccination against SARS-CoV-2 and its impact on severity and mortality caused by COVID-19. A previous study in Nicaragua had demonstrated that 78.5% of a sample of the Nicaraguan population had antibody titers against SARS-CoV-2. The present study was conducted with the aim of estimating the impact that vaccination had on severity and mortality in hospitalized patients with a diagnosis of COVID-19 in the Hospital Dr. Fernando Vélez Paiz, a referral hospital in Nicaragua.

**Methods:**

This is a retrospective cohort study. A total of 510 patients diagnosed with COVID-19 were hospitalized in the respiratory ward of the Hospital Dr. Fernando Vélez Paiz between January 2022 and October 2022. After applying the inclusion criteria, 220 patients were analyzed in the study. For the purpose of the study, the population that received at least one dose of vaccine against SARS-CoV-2 was denominated vaccinated. The national database of immunization was checked. The primary outcomes to evaluate were severity of illness (scores qCOVID, NEWS, and CXR), and mortality in vaccinated and unvaccinated populations. An analysis of risk relative (RR) in the exposed population (vaccinated) was done.

**Results:**

The mean age of the population was 67.2 ± 15.6 years old. Of the 220 patients, 157 (71.4%) had received at least one dose of the vaccine, and 63 (38.6%) had not been vaccinated. The most frequent vaccine recorded was ChAdOx1 (Astra Zeneca or CoviShield), in 102 patients (65%) (Table 1). There was no difference in severity of illness at admission in the qCOVID score, NEWS score, and CXR score. There were 47 patients (21.4%) who died. The mortality rates in the unvaccinated and vaccinated patients were 30.2% and 17.8%, respectively (Table 2). The RR (CI95%) for mortality in the vaccinated patients was 0.59 (0.35-0.97), showing that vaccination is a significant factor for protection against the probability of dying. The Kaplan-Meier analysis shows higher chance of survival in vaccinated patients (Figure 1).

**Table 1.**
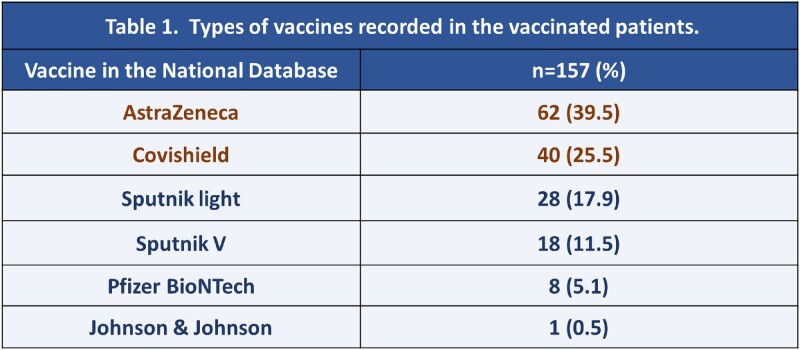
Types of Vaccines Recorded in Patients Hospitalized with COVID-19

**Table 2.**
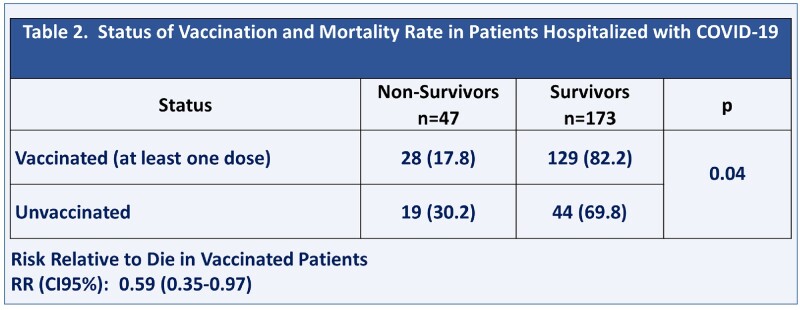
Mortality in Vaccinated and Unvaccinated Patients and Risk Relative for Mortality

**Figure 1.**
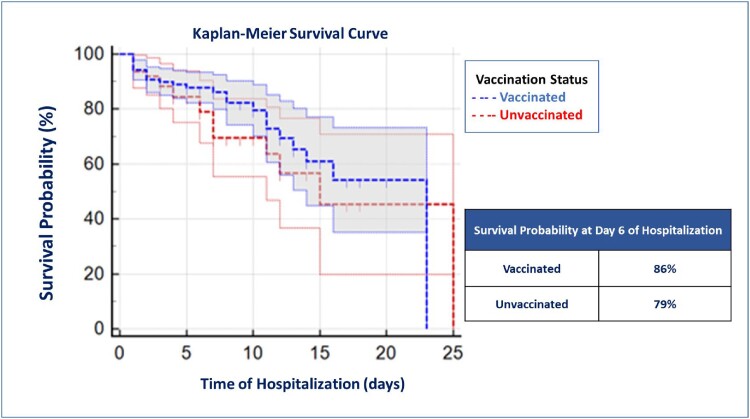
Survival Curve of Vaccinated and Unvaccinated Patients with COVID-19

**Conclusion:**

This study demonstrated that the mortality rate was lower in vaccinated versus non-vaccinated patients that were hospitalized in a respiratory ward due to COVID-19 in a hospital in Nicaragua.

**Disclosures:**

**All Authors**: No reported disclosures

